# Retraction: Functional analyses of micoRNA-326 in breast cancer development

**DOI:** 10.1042/BSR-2019-0787_RET

**Published:** 2021-07-28

**Keywords:** Breast cancer, miR-326, proliferation, SOX12

This article is being retracted from *Bioscience Reports* at the request of the authors following receipt of a notification from a reader, alerting the authors and Editorial Office to duplications in the Western blots of Figure 4D and 5B which have been published in several other publications by different authors.

The authors have been unable to replicate the results and wish to retract the article. The Editor-in-Chief and Editorial Board agree with the Retraction.

